# Supplementary material for: Antibody responses induced by trivalent inactivated influenza vaccine among pregnant and non-pregnant women in Thailand: A matched cohort study
Source: PLoS One. 2021 Jun 9;16(6):e0253028. doi: 10.1371/journal.pone.0253028 (PMC8189519; doi:10.1371/journal.pone.0253028)
Supplement: S1 Table — (PDF) [file pone.0253028.s001.pdf]

Supplemental Table 1. Vaccine strain matching during the study period*

Influenza strains	2018 TIV composition	Circulating strains	Match during the study period
**A(H1N1)**	A/Michigan/45/2015 (H1N1)pdm09	A/Michigan/45/2015 (H1N1)pdm09	100%
**A(H3N2)**	A/Singapore/ INFIMH-16-0019/2016 (H3N2)	A/Singapore/ INFIMH-16-0019/2016 (H3N2)<br>A/HongKong/4801/2014 (H3N2)	78.2%
**B**	B/Phuket /3073/2013 (Yamataga lineage)	B/Phuket /3073/2013 (Yamataga lineage)	100%

**Circulating strains and vaccine strain match data obtained from the sentinel surveillance of Thai National Institute of Health (August 2018 data)*
