# Supplementary material for: Antibody responses induced by trivalent inactivated influenza vaccine among pregnant and non-pregnant women in Thailand: A matched cohort study
Source: PLoS One. 2021 Jun 9;16(6):e0253028. doi: 10.1371/journal.pone.0253028 (PMC8189519; doi:10.1371/journal.pone.0253028)
Supplement: Attachment — Submitted filename: Response to Reviewers.docx [file pone.0253028.s003.docx]

**Response to Reviewers’ comments**

Dear Dr. Borrow,

We would like to thank you and the reviewers for reviewing our submission and providing us with valuable suggestions to improve the manuscript.

We have incorporated the reviewer’s feedback into our revised version of the manuscript. We have outlined our response to the journal comments and the specific comments of the reviewers and the changes made in the following section:

**Journal Requirements:**

1) Please review your reference list to ensure that it is complete and correct. If you have cited papers that have been retracted, please include the rationale for doing so in the manuscript text, or remove these references and replace them with relevant current references. Any changes to the reference list should be mentioned in the rebuttal letter that accompanies your revised manuscript. If you need to cite a retracted article, indicate the article’s retracted status in the References list and also include a citation and full reference for the retraction notice.

Author response:

We have rechecked each of the citations on PubMed and also the publisher websites for any retractions. We did not detect any of the cited papers to have been retracted. Kindly let us know if any of the cited papers have indeed been retracted, we will remove them from the reference list.

In response to the reviewer comments, we have made some changes to the references. Specifically, we have replaced the previous reference no. 7 with Domnich A, Manini I, Panatto D, Calabrò GE, Montomoli E. Immunogenicity measures of influenza vaccines: a study of 1164 registered clinical trials. Vaccines (Basel). 2020;8(2):325. doi: 10.3390/vaccines8020325.

We also added three more references (34-36) to address the comment of reviewer #1 on egg-adaptive mutations of A(H3N2):

34. Skowronski DM, Janjua NZ, De Serres G, Sabaiduc S, Eshaghi A, Dickinson JA, et al. Low 2012–13 influenza vaccine effectiveness associated with mutation in the egg-adapted H3N2 vaccine strain not antigenic drift in circulating viruses. PLoS One. 2014;9(3):e92153. doi: 10.1371/journal.pone.0092153.

35. Kissling E, Pozo F, Buda S, Vilcu A-M, Rizzo C, Gherasim A, et al. Effectiveness of influenza vaccine against influenza A in Europe in seasons of different A(H1N1)pdm09 and the same A(H3N2) vaccine components (2016–17 and 2017–18). Vaccine X. 2019;3:100042. doi: 10.1016/j.jvacx.2019.100042.

36. Cobey S, Gouma S, Parkhouse K, Chambers BS, Ertl HC, Schmader KE, et al. Poor immunogenicity, not vaccine strain egg adaptation, may explain the low H3N2 influenza vaccine effectiveness in 2012–2013. Clin Infect Dis. 2018;67(3):327-33. doi: 10.1093/cid/ciy097.

2) Please ensure that your manuscript meets PLOS ONE's style requirements, including those for file naming. The PLOS ONE style templates can be found at

https://journals.plos.org/plosone/s/file?id=wjVg/PLOSOne_formatting_sample_main_body.pdf and

https://journals.plos.org/plosone/s/file?id=ba62/PLOSOne_formatting_sample_title_authors_affiliations.pdf

Author response:

We have made some formatting changes to the title page and the body of the paper to meet PLOS ONE's style requirements. We have also edited the filenames as instructed in the templates.

3) Please provide an amended statement that declares *all* the funding or sources of support (whether external or internal to your organization) received during this study, as detailed online in our guide for authors at http://journals.plos.org/plosone/s/submit-now. Please also include the statement “There was no additional external funding received for this study.” in your updated Funding Statement.

Please include your amended Funding Statement within your cover letter. We will change the online submission form on your behalf.

Author response:

We have updated the Funding Statement as suggested to indicate only one funding source and that there was no additional external funding. We have also included our amended Funding Statement in our new cover letter.

4) While revising your submission, please upload your figure files to the Preflight Analysis and Conversion Engine (PACE) digital diagnostic tool, https://pacev2.apexcovantage.com/. PACE helps ensure that figures meet PLOS requirements. Please note that Supporting Information files do not need this step.

Author response:

We have now used the PACE tool to convert our figures to meet PLOS requirements and resubmitted them along with this revised version.

**Reviewer comments:**

**Reviewer #1:** This is a carefully done study and the findings are of considerable interest. Findings of this paper suggest that pregnancy did not alter immune responses to trivalent inactivated influenza vaccine (IIV3).

A few minor revisions are listed below.

1. In this paper, it is written that the highest sero-protection was found against A(H3N2) at 92.6% among vaccinated non-pregnant participants. However, recently lower effectiveness or no effectiveness of inactivated vaccine against A/H3N2 strain is frequently reported. Accordingly, although immune responses of pregnant women are sufficient, actual effectiveness may be low. In the Discussion, authors should explain about this problem, egg-adapted H3N2 vaccine strain problem, although in this study, vaccine effectiveness was not tested.

Skowronski DM, et al. Low 2012-13 influenza vaccine effectiveness associated with mutation in the egg-adapted H3N2 vaccine strain not antigenic drift in circulating viruses. PLoS One. 2014;9(3):e92153.

Author response:

We would like to thank the reviewer for the suggestion. Like the reviewer mentioned, previous studies have reported low effectiveness against A(H3N2) in all age groups. While some authors have attributed the low VE to the egg propagation-derived antigenic mismatch of the vaccine seed virus with circulating strains, others have indicated that it might have been caused by low vaccine immunogenicity instead of the egg adaptation of the vaccine strain. Contrary to these studies, we found the IIV3 to be immunogenic against A(H3N2) and the findings of the larger cohort study found the IIV3 to be effective against A(H3N2) in the same population of pregnant women.

We have added this information in the discussion section (lines 332-336) in the revised manuscript.

2. In this paper, authors concluded that seasonal IIV3 was immunogenic against all three vaccine strains and pregnancy did not seem to alter the immune response to the IIV3. And authors wrote that larger cohort studies with supplementary markers of humoral and cellular immunity may be needed. It is right, but in Thailand, I think that vaccine effectiveness study in adults, including pregnant women, is probably more needed, by cohort study or by test-negative study

Author response:

We agree with the reviewer that influenza VE studies (cohort or test-negative design) are needed among pregnant women in Thailand. We have cited our one-year prospective cohort study which enrolled 1726 pregnant women in Nakhon Phanom and found significant VE against the three influenza strains. Larger studies with immune responses (humoral and cellular) and clinical correlations of effectiveness are warranted, especially to assess the effect of repeated vaccinations.

The conclusion was been edited slightly to reflect this information (lines 366-368).

**Reviewer #2:** The authors present results from a matched cohort study in Thailand focused on antibody responses induced by the trivalent influenza vaccine (IIV3) among pregnant and non-pregnant women. They find that vaccinated women (both pregnant and non-pregnant) exhibited stronger antibody responses at 1 month than non-vaccinated women and conclude that pregnancy does not alter the response to the vaccine. The manuscript will be strengthened if the authors consider the following points:

1. More detail should be provided for the multilevel models used for analysis. Presumably a different model (though still within the broader class of multilevel models) was used for seroconversion or seroprotection than for the continuous antibody response variables. Authors should clarify the specific model(s) used, which variables or factors were included, including any interactions of interest, and information about any random effects utilized in the models.

Author response:

We have added the details of the multilevel models used in the study (lines 184-191). Specifically, we used mixed-effects linear regression model for the multilevel analyses of GMT and GMR, and mixed-effects negative binomial regression model for seroprotection and seroconversion. In all the analyses, we kept the interaction terms between pregnancy status and IIV3 vaccination status, controlled the variables of previous year vaccination history and baseline antibody levels, and used IIV3 vaccination status as the random intercept.

2. Throughout the manuscript (lines 40, 48, 202, 219, 230, 249, 253, 258) authors use "similar" or "comparable" when presenting results with a p-value>0.05. Authors should be careful in their terminology, as a conclusion of not rejecting the null hypothesis (p>0.05) does not mean groups are similar or comparable - this just means there is insufficient evidence in the data to support a hypothesis that they are different. This is especially important given the small sample sizes in the groups. A test to evaluate whether groups are "similar" or "equivalent" is different than one used to evaluate whether groups are different. One possible change would be to replace "similar" or "comparable" with "not significantly different" (or modified appropriately in the context of the sentence).

Author response:

We thank the reviewer for flagging this important distinction. We agree that the use of words such as “similar” and “comparable” is not valid in our case. We have changed the language in the aforementioned lines to now read “not significantly different” (lines 49-50, 58, 215, 217, 233-234, 244-245, 262-263, 268, 271).

3. STATA should be changed to Stata (lines 163, 182) - https://www.statalist.org/forums/help#spelling

Author response:

We have changed the spelling to now read “Stata” (lines 171, 195).

**Reviewer #3:** This is an interesting and well-written article describing antibody responses in pregnant and non-pregnant women in rural Thailand. Though studies are sometimes deemed less influential when no differences are found, I believe this study will have an important impact by demonstrating the high antibody titers induced in both pregnant and non-pregnant women.

The authors elaborated on the concerns regarding immunogenicity and clinical correlation on page 16 in the Discussion, but the Introduction includes a sentence that "immunogenicity of influenza vaccine is an important measure of vaccine effectiveness." This statement made me question the results and should be tempered by the later discussion points.

Additionally, I think it is important to recognize that immunogenicity data helps us understand vaccine responses in different populations and may be used for more than physician recommendations.

The Methods section is clear and concise. I appreciated that pregnant participants were matched by age group, gestational age, and week of vaccination to prevent undue influence from these factors. I also valued the contribution of study nurses who were able to travel to participants' homes to obtain blood samples when participants were unable to travel.

The Results section is also clear and easy to follow. I find it very interesting that so few women received influenza vaccine in the previous year, and I think this may at least partially explain the excellent responses to vaccination. The authors do an excellent job of comparing and contrasting these responses to Schlaudecker et al.'s study in the Discussion section. The previous vaccination may be an interesting idea for future research, especially if some of these women end of receiving more influenza vaccines in the future. I also valued the thorough discussion of HI titers and clinical correlation, as this is a growing area of concern. The authors appropriately mention future areas of study, such as neutralization titers and plasmablasts.

All in all, this article makes an important contribution to the understanding of influenza vaccination in pregnant women, which will likely increase in importance as more vaccines are recommended during pregnancy.

Author response:

We wholeheartedly thank the reviewer for the kind feedback and appreciation.

We have addressed the concern of the reviewer in the Introduction by editing the sentence "immunogenicity of influenza vaccine is an important measure of vaccine effectiveness" to emphasize immunogenicity as a measure of how well the vaccine responds in different populations. We have removed the previous point of provider recommendations from the sentence and changed the reference accordingly.

We hope that our changes will have addressed all the comments and meet with the approval of the reviewers.

Thank you for your kind consideration and we look forward to hearing from you soon.

Best regards,

**Manash Shrestha,**

Corresponding Author
