# Supplementary material for: Antibody responses induced by trivalent inactivated influenza vaccine among pregnant and non-pregnant women in Thailand: A matched cohort study
Source: PLoS One. 2021 Jun 9;16(6):e0253028. doi: 10.1371/journal.pone.0253028 (PMC8189519; doi:10.1371/journal.pone.0253028)
Supplement: Peer review history

# Peer review: Decision Letter 0

**DOI:** 10.1371/journal.pone.0253028.r001

**Authors:** Ray Borrow

4 May 2021

PONE-D-21-10606

Antibody responses induced by trivalent inactivated influenza vaccine among pregnant and non-pregnant women in Thailand: a matched cohort study

PLOS ONE

Dear Dr. Shrestha,

Thank you for submitting your manuscript to PLOS ONE. After careful consideration, we feel that it has merit but does not fully meet PLOS ONE’s publication criteria as it currently stands. Therefore, we invite you to submit a revised version of the manuscript that addresses the minor points raised below during the review process.

Please submit your revised manuscript by Jun 13 2021 11:59PM. If you will need more time than this to complete your revisions, please reply to this message or contact the journal office at plosone@plos.org. When you're ready to submit your revision, log on to https://www.editorialmanager.com/pone/ and select the 'Submissions Needing Revision' folder to locate your manuscript file.

Please include the following items when submitting your revised manuscript:

- A rebuttal letter that responds to each point raised by the academic editor and reviewer(s). You should upload this letter as a separate file labeled 'Response to Reviewers'.
- A marked-up copy of your manuscript that highlights changes made to the original version. You should upload this as a separate file labeled 'Revised Manuscript with Track Changes'.
- An unmarked version of your revised paper without tracked changes. You should upload this as a separate file labeled 'Manuscript'.

If you would like to make changes to your financial disclosure, please include your updated statement in your cover letter. Guidelines for resubmitting your figure files are available below the reviewer comments at the end of this letter.

If applicable, we recommend that you deposit your laboratory protocols in protocols.io to enhance the reproducibility of your results. Protocols.io assigns your protocol its own identifier (DOI) so that it can be cited independently in the future. For instructions see: http://journals.plos.org/plosone/s/submission-guidelines#loc-laboratory-protocols. Additionally, PLOS ONE offers an option for publishing peer-reviewed Lab Protocol articles, which describe protocols hosted on protocols.io. Read more information on sharing protocols at https://plos.org/protocols?utm_medium=editorial-email&utm_source=authorletters&utm_campaign=protocols.

We look forward to receiving your revised manuscript.

Kind regards,

Ray Borrow, Ph.D., FRCPath

Academic Editor

PLOS ONE

Journal Requirements:

1) Please review your reference list to ensure that it is complete and correct. If you have cited papers that have been retracted, please include the rationale for doing so in the manuscript text, or remove these references and replace them with relevant current references. Any changes to the reference list should be mentioned in the rebuttal letter that accompanies your revised manuscript. If you need to cite a retracted article, indicate the article’s retracted status in the References list and also include a citation and full reference for the retraction notice.

2)  Please ensure that your manuscript meets PLOS ONE's style requirements, including those for file naming. The PLOS ONE style templates can be found at

https://journals.plos.org/plosone/s/file?id=wjVg/PLOSOne_formatting_sample_main_body.pdf and

https://journals.plos.org/plosone/s/file?id=ba62/PLOSOne_formatting_sample_title_authors_affiliations.pdf

3)  Thank you for stating in your Funding Statement:

 [Yes - This study was partially supported by the Nakhon Phanom Provincial Hospital

Foundation (ref no. NP 0032.202.3/7) secured by KP and SN. The funders had no role

in study design, data collection and analysis, decision to publish, or preparation of the

manuscript.]. 

Please provide an amended statement that declares *all* the funding or sources of support (whether external or internal to your organization) received during this study, as detailed online in our guide for authors at http://journals.plos.org/plosone/s/submit-now.  Please also include the statement “There was no additional external funding received for this study.” in your updated Funding Statement.

Please include your amended Funding Statement within your cover letter. We will change the online submission form on your behalf.

[Note: HTML markup is below. Please do not edit.]

Reviewers' comments:

Reviewer's Responses to Questions

**Comments to the Author**

1. Is the manuscript technically sound, and do the data support the conclusions?

The manuscript must describe a technically sound piece of scientific research with data that supports the conclusions. Experiments must have been conducted rigorously, with appropriate controls, replication, and sample sizes. The conclusions must be drawn appropriately based on the data presented. 

Reviewer #1: Yes

Reviewer #2: Yes

Reviewer #3: Yes

**********

2. Has the statistical analysis been performed appropriately and rigorously? 

Reviewer #1: I Don't Know

Reviewer #2: Yes

Reviewer #3: Yes

**********

3. Have the authors made all data underlying the findings in their manuscript fully available?

The PLOS Data policy requires authors to make all data underlying the findings described in their manuscript fully available without restriction, with rare exception (please refer to the Data Availability Statement in the manuscript PDF file). The data should be provided as part of the manuscript or its supporting information, or deposited to a public repository. For example, in addition to summary statistics, the data points behind means, medians and variance measures should be available. If there are restrictions on publicly sharing data—e.g. participant privacy or use of data from a third party—those must be specified.

Reviewer #1: Yes

Reviewer #2: Yes

Reviewer #3: Yes

**********

4. Is the manuscript presented in an intelligible fashion and written in standard English?

PLOS ONE does not copyedit accepted manuscripts, so the language in submitted articles must be clear, correct, and unambiguous. Any typographical or grammatical errors should be corrected at revision, so please note any specific errors here.

Reviewer #1: Yes

Reviewer #2: Yes

Reviewer #3: Yes

**********

5. Review Comments to the Author

Please use the space provided to explain your answers to the questions above. You may also include additional comments for the author, including concerns about dual publication, research ethics, or publication ethics. (Please upload your review as an attachment if it exceeds 20,000 characters)

Reviewer #1: This is a carefully done study and the findings are of considerable interest. Findings of this paper suggest that pregnancy did not alter immune responses to trivalent inactivated influenza vaccine (IIV3).

A few minor revisions are listed below.

In this paper, it is written that the highest sero-protection was found against A(H3N2) at 92.6% among vaccinated non-pregnant participants. However, recently lower effectiveness or no effectiveness of inactivated vaccine against A/H3N2 strain is frequently reported. Accordingly, although immune responses of pregnant women are sufficient, actual effectiveness may be low. In the Discussion, authors should explain about this problem, egg-adapted H3N2 vaccine strain problem, although in this study, vaccine effectiveness was not tested.

Skowronski DM, et al. Low 2012-13 influenza vaccine effectiveness associated with mutation in the egg-adapted H3N2 vaccine strain not antigenic drift in circulating viruses. PLoS One. 2014;9(3):e92153.

In this paper, authors concluded that seasonal IIV3 was immunogenic against all three vaccine strains and pregnancy did not seem to alter the immune response to the IIV3. And authors wrote that larger cohort studies with supplementary markers of humoral and cellular immunity may be needed.It is right, but in Thailand, I think that vaccine effectiveness study in adults, including pregnant women, is probably more needed, by cohort study or by test-negative study.

Reviewer #2: The authors present results from a matched cohort study in Thailand focused on antibody responses induced by the trivalent influenza vaccine (IIV3) among pregnant and non-pregnant women. They find that vaccinated women (both pregnant and non-pregnant) exhibited stronger antibody responses at 1 month than non-vaccinated women and conclude that pregnancy does not alter the response to the vaccine. The manuscript will be strengthened if the authors consider the following points:

1. More detail should be provided for the multilevel models used for analysis. Presumably a different model (though still within the broader class of multilevel models) was used for seroconversion or seroprotection than for the continuous antibody response variables. Authors should clarify the specific model(s) used, which variables or factors were included, including any interactions of interest, and information about any random effects utilized in the models.

2. Throughout the manuscript (lines 40, 48, 202, 219, 230, 249, 253, 258) authors use "similar" or "comparable" when presenting results with a p-value>0.05. Authors should be careful in their terminology, as a conclusion of not rejecting the null hypothesis (p>0.05) does not mean groups are similar or comparable - this just means there is insufficient evidence in the data to support a hypothesis that they are different. This is especially important given the small sample sizes in the groups. A test to evaluate whether groups are "similar" or "equivalent" is different than one used to evaluate whether groups are different. One possible change would be to replace "similar" or "comparable" with "not significantly different" (or modified appropriately in the context of the sentence).

3. STATA should be changed to Stata (lines 163, 182) - https://www.statalist.org/forums/help#spelling

Reviewer #3: This is an interesting and well-written article describing antibody responses in pregnant and non-pregnant women in rural Thailand. Though studies are sometimes deemed less influential when no differences are found, I believe this study will have an important impact by demonstrating the high antibody titers induced in both pregnant and non-pregnant women.

The authors elaborated on the concerns regarding immunogenicity and clinical correlation on page 16 in the Discussion, but the Introduction includes a sentence that "immunogenicity of influenza vaccine is an important measure of vaccine effectiveness." This statement made me question the results and should be tempered by the later discussion points.

Additionally, I think it is important to recognize that immunogenicity data helps us understand vaccine responses in different populations and may be used for more than physician recommendations.

The Methods section is clear and concise. I appreciated that pregnant participants were matched by age group, gestational age, and week of vaccination to prevent undue influence from these factors. I also valued the contribution of study nurses who were able to travel to participants' homes to obtain blood samples when participants were unable to travel.

The Results section is also clear and easy to follow. I find it very interesting that so few women received influenza vaccine in the previous year, and I think this may at least partially explain the excellent responses to vaccination. The authors do an excellent job of comparing and contrasting these responses to Schlaudecker et al.'s study in the Discussion section. The previous vaccination may be an interesting idea for future research, especially if some of these women end of receiving more influenza vaccines in the future. I also valued the thorough discussion of HI titers and clinical correlation, as this is a growing area of concern. The authors appropriately mention future areas of study, such as neutralization titers and plasmablasts.

All in all, this article makes an important contribution to the understanding of influenza vaccination in pregnant women, which will likely increase in importance as more vaccines are recommended during pregnancy.

**********

6. PLOS authors have the option to publish the peer review history of their article (what does this mean?). If published, this will include your full peer review and any attached files.

If you choose “no”, your identity will remain anonymous but your review may still be made public.

**Do you want your identity to be public for this peer review?** For information about this choice, including consent withdrawal, please see our Privacy Policy.

Reviewer #1: No

Reviewer #2: No

Reviewer #3: No

[NOTE: If reviewer comments were submitted as an attachment file, they will be attached to this email and accessible via the submission site. Please log into your account, locate the manuscript record, and check for the action link "View Attachments". If this link does not appear, there are no attachment files.]

While revising your submission, please upload your figure files to the Preflight Analysis and Conversion Engine (PACE) digital diagnostic tool, https://pacev2.apexcovantage.com/. PACE helps ensure that figures meet PLOS requirements. To use PACE, you must first register as a user. Registration is free. Then, login and navigate to the UPLOAD tab, where you will find detailed instructions on how to use the tool. If you encounter any issues or have any questions when using PACE, please email PLOS at figures@plos.org. Please note that Supporting Information files do not need this step.

# Author comment: Author response to Decision Letter 0

**DOI:** 10.1371/journal.pone.0253028.r002

19 May 2021

Please find the response to reviewers in a separate word file attached with the submission.

Attachment Submitted filename: Response to Reviewers.docx Click here for additional data file.

# Editor report: Decision Letter 1

**DOI:** 10.1371/journal.pone.0253028.r003

**Authors:** Ray Borrow

27 May 2021

Antibody responses induced by trivalent inactivated influenza vaccine among pregnant and non-pregnant women in Thailand: a matched cohort study

PONE-D-21-10606R1

Dear Dr. Shrestha,

We’re pleased to inform you that your manuscript has been judged scientifically suitable for publication and will be formally accepted for publication once it meets all outstanding technical requirements.

Within one week, you’ll receive an e-mail detailing the required amendments. When these have been addressed, you’ll receive a formal acceptance letter and your manuscript will be scheduled for publication.

An invoice for payment will follow shortly after the formal acceptance. To ensure an efficient process, please log into Editorial Manager at http://www.editorialmanager.com/pone/, click the 'Update My Information' link at the top of the page, and double check that your user information is up-to-date. If you have any billing related questions, please contact our Author Billing department directly at authorbilling@plos.org.

If your institution or institutions have a press office, please notify them about your upcoming paper to help maximize its impact. If they’ll be preparing press materials, please inform our press team as soon as possible -- no later than 48 hours after receiving the formal acceptance. Your manuscript will remain under strict press embargo until 2 pm Eastern Time on the date of publication. For more information, please contact onepress@plos.org.

Kind regards,

Ray Borrow, Ph.D., FRCPath

Academic Editor

PLOS ONE

Additional Editor Comments (optional):

Reviewers' comments:

# Editor report: Acceptance letter

**DOI:** 10.1371/journal.pone.0253028.r004

**Authors:** Ray Borrow

31 May 2021

PONE-D-21-10606R1 

Antibody responses induced by trivalent inactivated influenza vaccine among pregnant and non-pregnant women in Thailand: a matched cohort study 

Dear Dr. Shrestha:

I'm pleased to inform you that your manuscript has been deemed suitable for publication in PLOS ONE. Congratulations! Your manuscript is now with our production department. 

If your institution or institutions have a press office, please let them know about your upcoming paper now to help maximize its impact. If they'll be preparing press materials, please inform our press team within the next 48 hours. Your manuscript will remain under strict press embargo until 2 pm Eastern Time on the date of publication. For more information please contact onepress@plos.org.

If we can help with anything else, please email us at plosone@plos.org. 

Thank you for submitting your work to PLOS ONE and supporting open access. 

Kind regards, 

PLOS ONE Editorial Office Staff

on behalf of

Prof. Ray Borrow 

Academic Editor

PLOS ONE
